# Assessment of Residual Solvent and Drug in PLGA Microspheres by Derivative Thermogravimetry

**DOI:** 10.3390/pharmaceutics12070626

**Published:** 2020-07-04

**Authors:** Hyunjin Shim, Hongkee Sah

**Affiliations:** College of Pharmacy, Ewha Womans University, 52 Ewhayeodaegil, Seodaemun-gu, Seoul 03760, Korea; jinshim@ewhain.net

**Keywords:** poly-*d,l*-lactide-*co*-glycolide, microspheres, derivative thermogravimetry, residual solvent, ethyl formate

## Abstract

Thermogravimetry does not give specific information on residual organic solvents in polymeric matrices unless it is hyphenated with the so-called evolved gas analysis. The purpose of this study was to apply, for the first time, derivative thermogravimetry (DTG) to characterize a residual solvent and a drug in poly-*d,l*-lactide-*co*-glycolide (PLGA) microspheres. Ethyl formate, an ICH class 3 solvent, was used to encapsulate progesterone into microspheres. DTG provided a distinct peak, displaying the onset and end temperatures at which ethyl formate started to evolve from to where it completely escaped out of the microspheres. DTG also gave the area and height of the solvent peak, as well as the temperature of the highest mass change rate of the microspheres. These derivative parameters allowed for the measurement of the amount of residual ethyl formate in the microspheres. Interestingly, progesterone affected not only the residual solvent amount but also these derivative parameters. Another intriguing finding was that there was a linear relationship between progesterone content and the peak height of ethyl formate. The residual solvent data calculated by DTG were quite comparable to those measured by gas chromatography. In summary, DTG could be an efficient and practical quality control tool to evaluate residual solvents and drugs in various polymeric matrices.

## 1. Introduction

Long-acting microspheres are leading dosage forms of injectable poly-*d,l*-lactide-*co*-glycolide (PLGA)-based drug delivery systems. PLGA microspheres providing sustained release of drugs over a wide range of periods can reduce dosing frequency, minimize drug side effects, and improve patient compliance. Emulsion-based solvent evaporation preparative techniques using methylene chloride or chloroform are commonly applied to prepare PLGA particles [1,2,3]. These solvents have low boiling points and negligible water-immiscibility. These features are advantageous to emulsion formation and solvent removal during a microsphere manufacturing process. However, using these solvents often brings up problematic issues such as particle aggregation, poor drug encapsulation efficiency, and non-uniform particle distribution. It is also of concern that methylene chloride and chloroform are ICH class 2 solvents that adversely affect environmental safety and human health. As an alternative organic solvent, ethyl acetate is often employed to produce micro- and nano-particles [4,5,6,7]. In the practice of a microsphere manufacturing process using ethyl acetate, solvent removal is usually achieved following the principle of solvent extraction. Sometimes solvent evaporation is used for microsphere hardening, but a long process time is required due to its low volatility [4].

The solvent type and its residual level in drug products and medical devices (e.g., microspheres, drug-eluting sutures, and polymer-coated stents) are strictly regulated by regulatory authorities. As far as solvent type is concerned, ICH class 3 solvents are preferred over ICH class 2 solvents in the pharmaceutical industry. Information on residual solvents in PLGA microspheres can be obtained by analytical tools such as loss on drying, thermogravimetry (TG), and gas chromatography (GC). At present, reliable methods of choice for identifying and quantifying residual solvents are gas chromatography (GC) or GC–mass spectrometry (GC–MS). In particular, dynamic headspace GC–MS provides excellent sensitivity, repeatability, and ease of sample preparative step. In a TG experiment, sample mass is monitored as a function of temperature as is subjected to a controlled temperature program. When the sample gives off volatile byproducts, TG provides useful information on the physical and chemical events it undergoes. Therefore, TG has found full applications in investigating the thermal stability of a material; the presence of volatile substances in an initial article; and its degradation temperature, chemical reactions, and inorganic residues. However, TG does not give specific information on the solvent type and its quantity, since it is a non-specific analytical method. Further, when a following thermal event overlaps any stages of a preceding thermal event, it is not easy to assess each event qualitatively or quantitatively. Therefore, in many studies of characterizing PLGA-based micro-/nano-particles through TG experiments, information on residual solvents was not thoroughly discussed [8,9,10,11]. To identify and quantify gas byproducts, TG should be combined with the so-called evolved gas analysis (EGA). Relevant techniques include TG–FTIR, TG–MS, and TG–GC–MS [12,13,14,15].

Derivative thermogravimetry (DTG) is a type of thermal analysis in which the rates of mass changes of a substance upon heating are plotted against temperature or time. Weight loss of a sample is first monitored over time using TG, and a DTG curve is derived from the TG curve. DTG makes it possible to obtain parameters related to a thermal event, such as its onset and end temperatures (To and Te), as well as the temperature at which the rate of mass change is highest (Tp). DTG has been used for thermal decomposition studies of various materials such as carbon spheres, polyaniline-cellulose nanofiber, laterite, and amyrenone [16,17,18,19]. DTG was also used to analyze the composition ratio of a polymer composite. For example, Guarino et al. prepared a poly(ε-caprolactone)-poly(L-lactide) composite (85:15) scaffold and analyzed its composition changes throughout degradation [20,21]. During pyrolysis, the two polymers underwent thermal decomposition at different temperatures. On the basis of this information, DTG was used to calculate the composition ratio of the scaffold. As far as long-acting PLGA microspheres are concerned, their thermal properties are predominantly characterized by differential scanning calorimetry (DSC) and TG [22,23]. However, to the best of our knowledge, DTG has not been applied to investigating the thermal behavior and quantification of a residual organic solvent and a drug in PLGA microspheres.

Ethyl formate, a non-halogenated ICH 3 class 3 solvent, has been recently proposed as an attractive dispersed solvent that can be used in the preparation of drug-containing PLGA microspheres [24]. It was demonstrated that the ethyl formate-based preparative technique permitted the manufacturing of PLGA microspheres with excellent quality attributes. Progesterone (log P = 3.87) is practically insoluble in water (8.8 μg/mL). Postmenopausal women are often treated with estrogen or estrogen with progesterone. On the basis of these facts, progesterone was chosen as a model hydrophobic drug that was subject to the ethyl formate-based microencapsulation process. For the first time, DTG was applied to assess residual ethyl formate in the microspheres. Further, DTG was used to evaluate the effect of progesterone on the thermal behavior of residual ethyl formate. The feasibility of DTG in quantifying the residual solvent was verified by a GC method. The primary purpose of this study was to apply DTG toward PLGA microspheres prepared using ethyl formate. Currently, most of PLGA microspheres are being manufactured using methylene chloride. Accordingly, it was deemed appropriate to evaluate whether or not our DTG technique could be extended toward PLGA microspheres prepared using methylene chloride. Residual methylene chloride data determined by DTG were compared to those analyzed by GC. Table 1 summarizes major physicochemical properties of ethyl formate and methylene chloride.

## 2. Materials and Methods

### 2.1. Materials

75:25DLG 4A (PLGA with a lactide/glycolide ratio of 75:25; inherent viscosity, 0.4 dL/g in CHCl_3_ at 25 °C) was supplied from Evonik Degussa Corp. (Birmingham, AL, USA). This polymer is abbreviated as PLGA in the text. Polyvinyl alcohol (PVA; 88% hydrolyzed, Mw = 25,000 g/mol) was obtained from Polysciences, Inc. (Warrington, PA, USA). Ethyl formate was from Sigma-Aldrich Korea (Seoul, Korea). Progesterone and methylene chloride were procured from TCI Chemicals (Tokyo, Japan) and Showa Denko (Tokyo, Japan), respectively.

### 2.2. Preparation of PLGA Microspheres Using Ethyl Formate

Progesterone-loaded PLGA microspheres were prepared following the microencapsulation method reported elsewhere [24]. Briefly, PLGA (250 mg) and progesterone (40, 70, 100, or 130 mg) were dissolved in 3 mL of ethyl formate. This dispersed phase was poured onto 25 mL of a 0.5% PVA aqueous solution that was being stirred at 450 rpm by a digital stirrer (model 400 series; VWR Scientific, Radnor, PA, USA). After 5 min stirring, 50 mL of a 0.1% PVA solution was added to the oil-in-water (*o/w*) emulsion, in order to convert emulsion droplets into embryonic microspheres. After 5 min elapsed, the embryonic microsphere suspension was poured into 100 mL of a 0.1% PVA solution and was subject to continual stirring at 450 rpm for 1 h. This microsphere suspension was passed through two sieves with 425 μm and 25 μm apertures. The microspheres caught between the two sieves were redispersed in 100 mL of the fresh 0.1% PVA solution, which was stirred at 30 °C for 3 h. This step was carried out to complete the hardening of microspheres. Microspheres were then collected by filtration and were vacuum-dried overnight at ambient temperature. Blank, progesterone-free microspheres were also prepared using the same manufacturing process. Microspheres with a specific formulation were prepared at least in triplicate.

### 2.3. Preparation of PLGA Microspheres Using Methylene Chloride

An emulsion-based solvent evaporation process was used to prepare PLGA microspheres. PLGA (250 mg) and progesterone (0 or 100 mg) were dissolved in 3 mL of methylene chloride. This dispersed phase was emulsified in 50 mL of a 0.5% PVA aqueous solution by using the digital stirrer described above. The appearing *o/w* emulsion was subjected to continuous stirring for 4 h at room temperature. A sieve with a pore size of 425 µm was placed on a sieve with a pore size of 25 µm, and then the microsphere suspension was poured into the top sieve. The microspheres present between the two sieves were collected, filtered, and vacuum-dried overnight.

### 2.4. Particle Size Analysis

The size distribution of PLGA microspheres was measured by a laser diffraction particle size analyzer (Mastersizer 3000E; Malvern Instruments Ltd., Worcestershire, UK). After the wet sieving described in the above microencapsulation processes finished, we collected the microspheres and diluted them with water to the final volume of 540 mL. The microsphere suspension was placed inside the sample measurement cell, and their volume-based size was determined.

### 2.5. Scanning Electron Microscopy (SEM)

The JSM-5200 scanning electron microscope (Jeol Inc., Tokyo, Japan) was used to examine the morphology of microspheres. They were sprinkled over a double-sided adhesive tape mounted on a metal specimen stub. Prior to examination with SEM, microspheres were coated by using a SC7620 sputter coater (VG Microtech, West Sussex, UK).

### 2.6. Encapsulation Efficiency of Progesterone

After microsphere samples (18.3–20.5 mg) were entirely dissolved in 4 mL of tetrahydrofuran, we added 16 mL of a methanol-water mixture (8:2, by *v/v*) to induce PLGA precipitation. The appearing PLGA precipitates were removed by using the Spin-X centrifuge filter with a pore size of 0.22 μm. The filtrate (10 μL) was injected into HPLC, as well as its progesterone, and its concentration in the filtrate was analyzed. The Luna C18 column (5 μm, 100 Å, 30 cm in length) was used as an analytical column at controlled room temperature. The methanol-water mixture (8:2, by *v/v*) was employed as a mobile phase at a flow rate of 0.8 mL/min. Progesterone was detected at 254 nm, and a standard calibration curve (45.4–712 μg/mL) was used to calculate its concentration. Percentage encapsulation efficiency (EE) of progesterone was calculated by the following equation:

Progesterone EE% = 100 × (actual drug loading/theoretical drug loading)(1)

where actual drug loading is (the amount of progesterone in a microsphere sample)/(the weight of a microsphere sample), and theoretical drug loading represents (the initial amount of progesterone used for microencapsulation)/(the initial amounts of progesterone and PLGA used for microencapsulation).

### 2.7. Gas Chromatography (GC)

Microsphere samples (26.4–33.1 mg) were wholly dissolved in 4 mL of tetrahydrofuran, to which 12 mL of methanol was added to induce PLGA precipitation. The Spin-X centrifuge filter was used to remove PLGA precipitates, and the filtrate was subjected to GC. After being spiked with an internal standard solution, an aliquot (1 μL) was injected into the Shimadzu GC 2010. The Zebron ZB-624 capillary column (30 m × 0.32 mm × 1.80 µm) was used as an stationary column, while nitrogen gas at a flow rate of 1.75 mL/min was employed as a carrier. The gas analytes eluting from the column were detected by FID. Quantitative analysis of ethyl formate was carried out using ethyl acetate as an internal standard (IS). Ethyl formate standard solutions of 5 different known concentrations was prepared using the methanol-tetrahydrofuran mixture. A constant amount (0.2 mL) of the IS solution was added to the sample solution (0.6 mL) and the ethyl formate standard solutions (0.6 mL). After GC analysis, both peak areas of ethyl formate and IS were computed. A calibration plot of the peak area ratios vs. ethyl formate concentrations was established to calculate the concentration of ethyl formate in the sample. Residual methylene chloride in microspheres was quantified in a similar way, using ethyl formate as an IS.

### 2.8. Thermogravimetry (TG)

Thermogravimetric analyses of PLGA raw powders, progesterone, and PLGA microspheres with various progesterone payloads were carried out using the Q50 thermogravimetric analyzer (TA Instruments; New Castle, DE, USA). Prior to a TG experiment, the system was calibrated using indium. Samples (10.01–18.84 mg) were put into a platinum pan. While the system was purged with nitrogen gas a flow rate of 40 mL/min, the pan was heated at a rate of 20 °C/min over a temperature range between 30 and 550 °C. Data were treated with the Universal Analysis software (version 4.5A).

### 2.9. Derivative Thermogravimetry (DTG)

A TG curve representing changes in percent mass of microspheres upon heating was derivatized in terms of temperature and time. This derivatization provided the data of *d*(% mass)/*d* (°C) and *d*(% mass)/*d*(min). After these data were plotted against temperature, we derived the thermal parameters. “To” was defined as the onset temperature in which ethyl formate started to evolve from PLGA microspheres. “Te” was the end temperature at which ethyl formate completely disappeared out of the microspheres. The peak temperature (“Tp”) represented the temperature of the maximum rate of mass loss. Coupling these derivative parameters to TG led to the calculation of the weight loss of microspheres caused by the residual solvent. The same DTG parameters (To, Te, and Tp) were also derived from PLGA microspheres prepared using methylene chloride.

## 3. Results and discussion

### 3.1. Characterization of PLGA Microspheres Prepared Using Ethyl Formate

**Figure 1:**
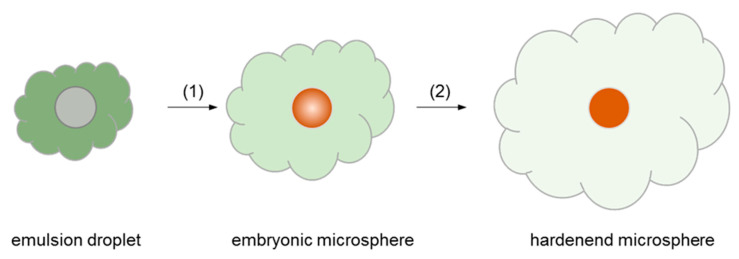
Pictorial representations of our essential microencapsulation approach. Step (1) is carried out to produce embryonic, semisolid microspheres. Step (2) stands for the primary solvent extraction process for microsphere hardening. The color gradient represents the difference in the aqueous ethyl formate concentration during microencapsulation. The fading intensity indicates that an aqueous ethyl formate concentration is being diluted, as the system is quenched with water.

Long-acting PLGA microsphere products containing hydrophobic drugs usually have drug payloads of around 20–35%. Zilretta, triamcinolone acetonide ER injection, has a drug load of 25%. Vivitrol contains 337 mg of naltrexone per gram of microspheres, while Signifor LAR is formulated with 34.2% of pasireotide pamoate. On consideration of these microsphere products, our microsphere formulations had theoretical progesterone payloads ranging from 13.8 to 34.2%. The microsphere manufacturing process made use of partial water miscibility and a low boiling point of ethyl formate. Figure 1 illustrates a schematic representation of a series of crucial unit processes. A critical step that most contributes to the successful preparation of microspheres is related to how to perform emulsification and solvent extraction. In our case, the aqueous to dispersed phase ratio was intentionally set at 25:3 in order to suppress the escape of ethyl formate from the dispersed phase into the aqueous phase. The use of a relatively small amount of the aqueous phase helped generate emulsion droplets and inhibit the formation of fiber-like PLGA agglomerates. Following this step, a small amount of extra water was added to convert emulsion droplets into embryonic microspheres. Subsequently, they were subjected to a full-fledged solvent extraction process.

**Figure 2:**
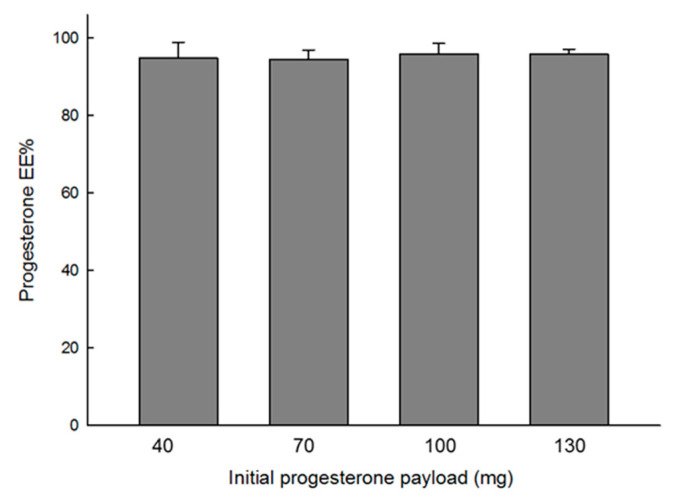
Effect of an initial progesterone amount used for microencapsulation upon its encapsulation efficiency (EE)%. The amount of poly-*d,l*-lactide-*co*-glycolide (PLGA) was fixed at 250 mg. The ethyl formate-based microencapsulation process provides almost complete encapsulation of progesterone into PLGA microspheres.

**Figure 3:**
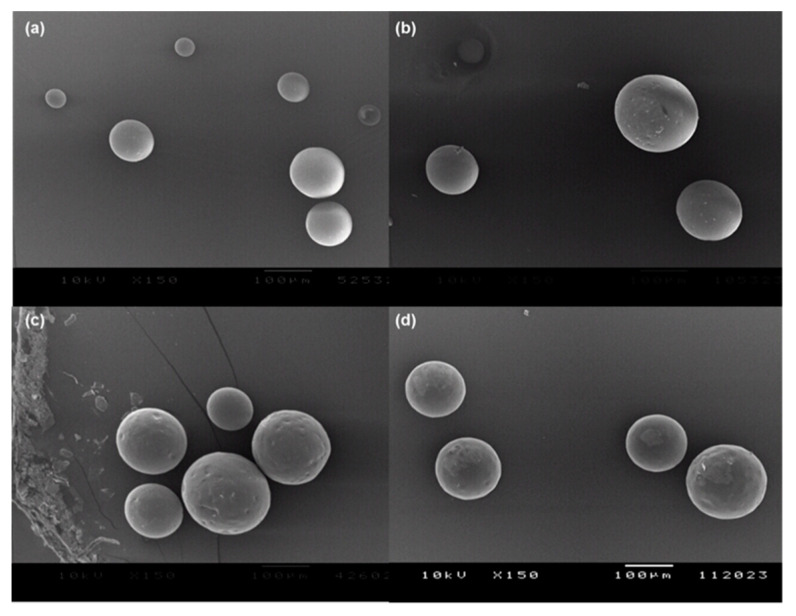
SEM micrographs of PLGA microspheres containing various amounts of progesterone. The actual drug payload was (**a**) 13.1%, (**b**) 20.7%, (**c**) 27.4%, or (**d**) 32.8%. The size bar is 100 μm.

When encapsulated into PLGA microspheres through our microencapsulation technique, progesterone was almost wholly loaded into PLGA microspheres (Figure 2). For example, when initial progesterone payloads were 40 and 130 mg, the corresponding drug EE values were 94.9 ± 4.0 and 95.8 ± 1.3%, respectively. Our EE results are far better than those previously reported by other research groups. In many cases of solvent evaporation processes using methylene chloride, unentrapped hydrophobic drug crystals tend to appear in microsphere suspensions. As a result, low encapsulation efficiencies are often observed. For example, when encapsulating risperidone in PLGA microspheres, its EE was as low as 14.4% [25]. In cases of cyclosporine A and norquetiapine, their EE values were 57.5 ± 3.0% and 48.3 ± 3.0%, respectively [26,27]. There are also a couple of studies encapsulating progesterone into PLGA microspheres via methylene chloride-based solvent evaporation methods [28,29]. Its EE values ranged from 78.2 to 84.6%. In our solvent evaporation process using methylene chloride, progesterone EE was determined to be 64.6 ± 2.6%. As mentioned above, the formation of unentrapped progesterone crystals was mainly attributed to the relatively low drug encapsulation. In comparison to those studies, the ethyl formate-based microsphere manufacturing process brought up excellent drug EE. As shown in Figure 3, the microspheres had spherical morphology, and no progesterone crystals were present on their non-porous and smooth surface. Figure S1 shows a typical size distribution of progesterone-containing PLGA microspheres. The morphology of PLGA microspheres prepared by solvent evaporation was also demonstrated in Figure S2.

### 3.2. Comparison of TG and DTG Curves of PLGA Microspheres

**Figure 4:**
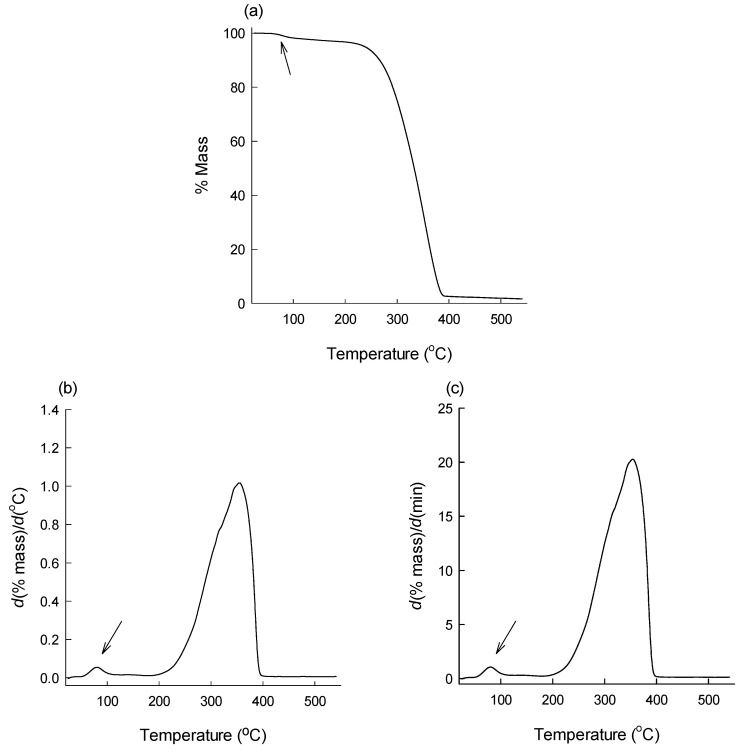
Typical (**a**) thermogravimetry (TG) and (**b**,**c**) derivative thermogravimetry (DTG) curves of PLGA microspheres. The first derivative of a TG curve was established with respect to (**b**) temperature and (**c**) time. A thermal event arising from the evolvement of residual ethyl formate in the microspheres is clearly demonstrated as the first peaks in the DTG curves.

**Figure 5:**
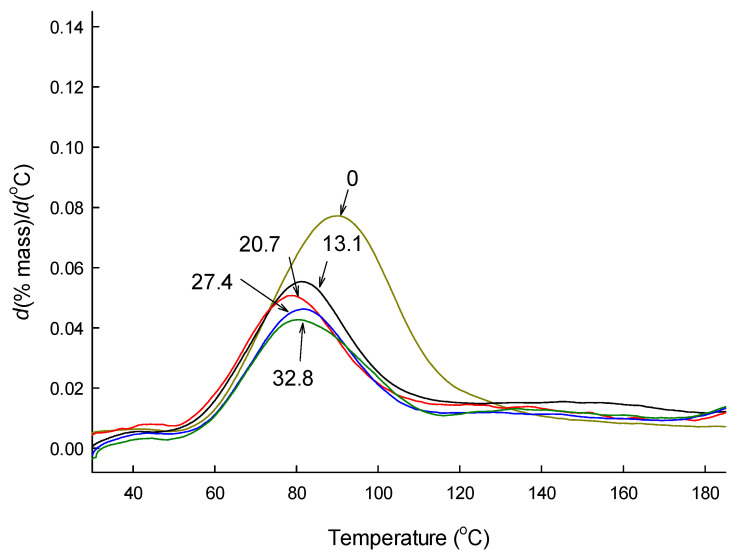
DTG curves of PLGA microspheres containing various amounts of progesterone. Numbers indicate actual progesterone payloads of different PLGA microspheres.

Figure 4 shows typical TG and DTG curves of a microsphere batch manufactured using ethyl formate. The TG curve illustrated that there was very little weight loss of microspheres below 200 °C, and the mass loss arising from the evolvement of ethyl formate was not sharply outlined (Figure 4a). On the contrary, its DTG curves displayed a clear peak indicative of the volatilization of residual ethyl formate in PLGA microspheres (Figure 4b,c). Figure 4 proves that DTG allows recognizing the thermal event of solvent evolvement more clearly than TG does. Moreover, DTG makes it easier to identify the temperatures at which the solvent volatilization begins and ends. Figure 5 illustrates portions of DTG curves of PLGA microspheres with various amounts of progesterone over the temperature range of 30 to 185 °C. Distinct peaks arising from the evolvement of ethyl formate from different microspheres appear clearly.

DTG curves show that the weight loss of microspheres stemming from residual ethyl formate begins at about 50 °C. This phenomenon is understandable, considering its boiling point of 54.4 °C. However, Te temperatures of all the microspheres were much higher than the boiling point of ethyl formate. For example, ethyl formate evolves continuously from progesterone-free PLGA microspheres up to 134.4 ± 3.0−138.8 ± 4.6 °C (Table 2). It is also notable that despite the fact that all microspheres displayed similar To values, their Te temperatures were vividly influenced by the amount of progesterone in microspheres. Blank microspheres showed the highest Te temperature compared with progesterone-loaded microspheres. Most previous DTG experiments that have been attempted for thermal decomposition studies of various materials opted to use the first derivative of *d*(% mass)/*d*(min). Our present DTG analysis derived not only *d*(% mass)/*d*(min) but also *d*(% mass)/*d*(°C). When compared, their results were quite similar to each other (Table 2).

### 3.3. Measurement of Residual Solvents by DTG

**Figure 6:**
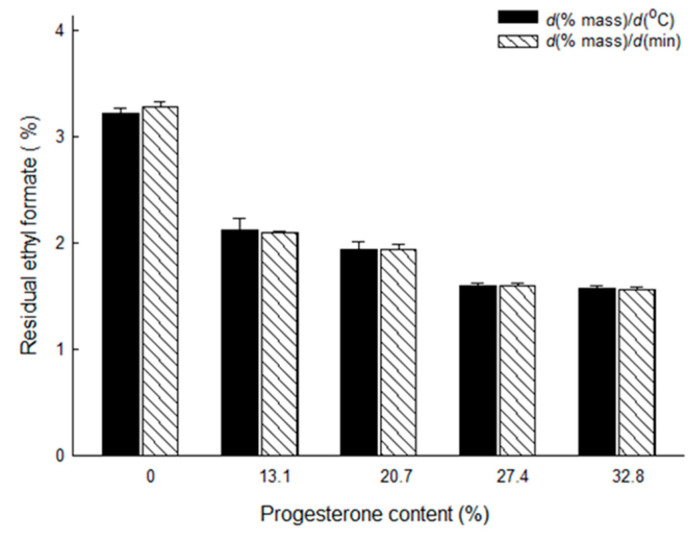
Amounts of residual ethyl formate in PLGA microspheres with different progesterone payloads. The first derivatives of a TG curve with respect to degrees Celsius and minutes were employed to calculate the content of residual ethyl formate.

**Figure 7:**
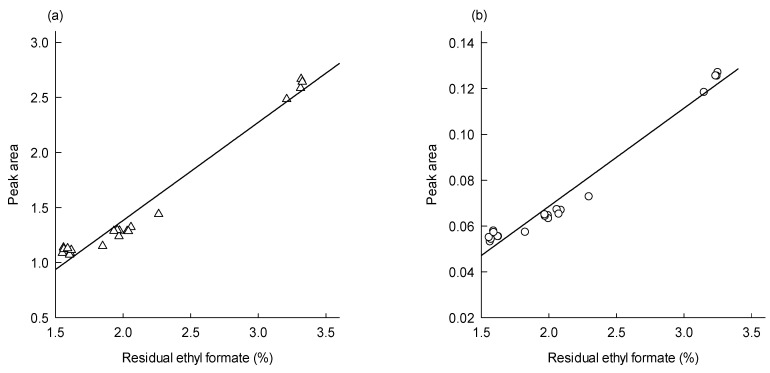
Linearity between the content of residual ethyl formate and the solvent peak area. The first derivative data with respect to (**a**) minutes and (**b**) degrees Celsius were utilized to compute the peak area of ethyl formate.

Residual ethyl formate in PLGA microspheres was determined by subtracting a microsphere weight at Te from its weight at To. Figure 6 shows the levels of residual ethyl formate in PLGA microspheres with different progesterone payloads. Residual solvent data determined by the first derivative data of *d*(% mass)/*d*(°C) were quite comparable to those measured by the first derivative data of *d*(% mass)/*d*(min). Additionally, it was of interest to notice that the level of residual ethyl formate was substantially influenced by progesterone content in microspheres. Progesterone-free microspheres contained 3.22 ± 0.05–3.29 ± 0.05% of ethyl formate, but its residual content in PLGA microspheres with 32.8% of progesterone was 1.58 ± 0.02%. Residual ethyl formate contents calculated by our DTG technique were compared to those determined by the GC method. Figure S3 shows a typical GC chromatogram of a microsphere sample and a standard calibration curve. As shown in Table 3, both assay methods gave quite similar results. For example, when the microspheres containing 32.8% progesterone were analyzed by GC and DTG methods, the difference in their mean values of residual ethyl formate were not statistically different (*p* ≥ 0.076). The geometric GC/DTG mean ratio value ranged from 0.95 to 1.03. Another intriguing fact revealed through this study was that there was a linear relationship between the peak area of ethyl formate and its residual amount in microspheres (Figure 7). A preferred level of ICH class 3 solvents in drug products is below 0.5%. In the future, our ethyl formate-based preparative technique will be improved to reduce its residual amount in PLGA microspheres to below 0.5%. When this challenge is succeeded, the resultant peak areas of ethyl formate will be included in the straight line shown in Figure 7.

It is often presumed that the point at which the weight loss of microspheres attributed to the volatilization of a residual organic solvent occurs around its boiling point [30]. For example, Javiya and Jonnalagadda prepared PLGA/PEG microspheres by spray-drying using methylene chloride. When subjected to TG, the microspheres did not display significant weight loss at 37 °C. On the basis of this observation, it was concluded that their microspheres did not contain residual methylene chloride [31]. However, our study strongly indicates that the escape of methylene chloride from polymeric dosage forms is likely to occur over a wide temperature range that exceeds its boiling point. It should also be emphasized that appropriate knowledge of To and Te temperatures is a prerequisite for the accurate measurement of a residual solvent in microspheres. However, TG does not provide such information. The following case was taken as an example to discuss this aspect. Manson and Dixon prepared a thin PLGA film by a solvent casting technique using methylene chloride [32]. The amount of residual methylene chloride in the 200 μm thick film was calculated as a weight loss between 80 and 200 °C through a TG experiment. However, the rationale for establishing such a temperature range was not justified.

**Figure 8:**
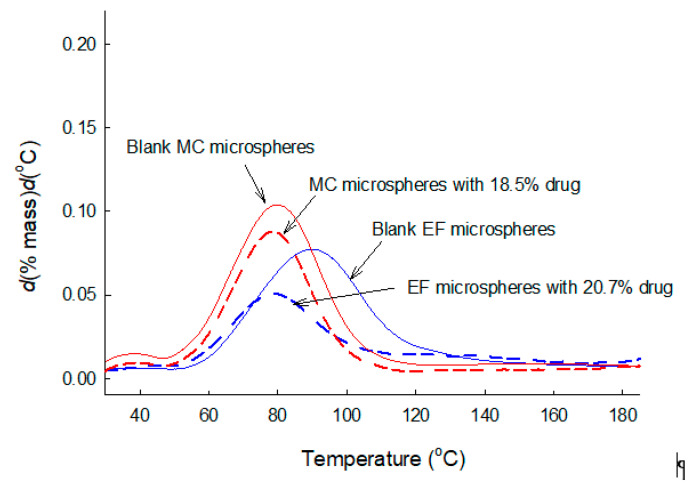
(Red) DTG curves of blank PLGA microspheres and those with 18.5% of progesterone. Methylene chloride (MC) was used for microencapsulation. (Blue) DTG curves of PLGA microspheres containing 0 or 20.7% of progesterone. Microspheres were prepared using ethyl formate (EF) as a dispersed solvent.

Our DTG study was expanded to challenge the above perception. PLGA microspheres were prepared by the methylene chloride-based solvent evaporation process, and the thermal behavior and content of residual methylene chloride were evaluated (Figure 8). The To and Te of blank microspheres were 49.1 ± 1.3 and 109.0 ± 3.3 °C. These values are higher than its boiling point (40 °C). The amount of residual methylene chloride measured by DTG was 3.51 ± 0.20%. Unlike the case of PLGA microspheres prepared using ethyl formate, neither To (48.4 ± 1.2 °C) nor Te (107.2 ± 1.9 °C) was significantly affected by the presence of 18.5% of progesterone in PLGA microspheres. However, the amount of residual methylene chloride in the progesterone-containing microspheres was reduced to 2.60 ± 0.38%. In all cases where microspheres were prepared using either ethyl formate or methylene chloride, their residual amounts tended to decrease when progesterone was present in the microsphere matrices. A GC experiment was also carried out to measure residual methylene chloride. A typical GC chromatogram and a standard calibration curve were illustrated in Figure S3. As seen in Table 4, residual methylene chloride data determined by DTG was quite comparable to those measured by GC. A statistical analysis proved that the difference in the mean vales of the two methods was not statistically significant (*p* ≥ 0.152). Our results support the possibility of DTG being applied to the assessment of the thermal behavior and quantities of various organic solvents residing in polymeric matrices.

### 3.4. Effect of Progestrone upon Thermal Behavior of Ethyl Formate

**Figure 9:**
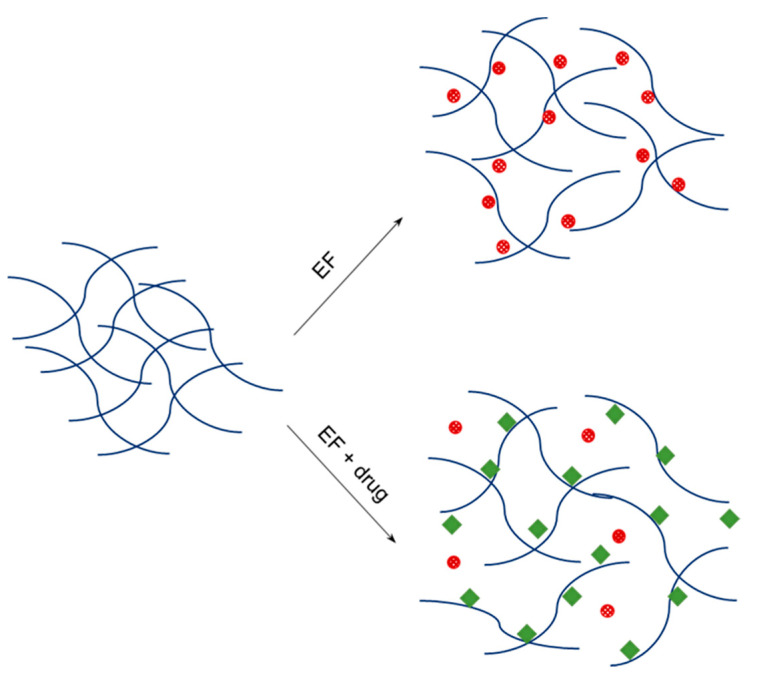
Interactions of ethyl formate (EF) and progesterone with PLGA polymer chains. Progesterone displaces ethyl formate from PLGA polymer chains or acts as a plasticizer to help ethyl formate escape relatively easily.

Several reports discussed drug–PLGA interactions but reached different conclusions. For example, when leuprorelin acetate was loaded into PLGA microspheres, basic amino acid residues in the peptide interacted with terminal carboxylic ends of PLGA chains, which increased the rigidity of the polymer [33]. As a result, the Tg of leuprorelin-containing PLGA microspheres increased gradually from 42 to 47 °C, with its payload rising from 0 to 8%. Noviendri et al. performed a TG experiment to suggest that there were no strong chemical interactions between fucoxanthin and the polymeric matrix of PLGA microspheres [10]. Most relevant studies, however, did not take into account the inter-relationships between residual solvent, drug, and PLGA. An interesting fact can be deduced when the results of Figure 6 and Table 2 are considered together—blank microspheres contain the highest level of residual ethyl formate and display the broadest To–Te breadth. The level of residual ethyl formate is pronouncedly reduced when progesterone is present in microspheres. Furthermore, progesterone helps shorten the To–Te range. Given these phenomena to deliberation, one could argue the hypothesis shown in Figure 9. Ethyl formate inside blank PLGA microspheres may be firmly bound to the microsphere matrix. In addition, the non-porous microsphere matrix suppresses the evolvement of ethyl formate entrapped deeply inside the microspheres. Consequently, its complete removal from the microspheres takes place at a temperature much higher than its boiling point. However, the strong PLGA polymer chains–ethyl formate interaction is likely to become loose in the presence of progesterone, due to the latter’s preferential interaction with the polymer chains. Another mechanism might be associated with the plasticizing effect of a drug upon PLGA polymers. For example, it was suggested elsewhere that the hydrogen bonding between ketoprofen and PLGA led to disruption of polymer chain–chain interactions, contributing to a decrease in the polymer Tg and an increase in chain mobility [34]. Likewise, progesterone serving as a plasticizer increases the flexibility and mobility of PLGA chains. Accordingly, the removal of residual ethyl formate becomes more comfortable if progesterone molecules are present in the microsphere matrix. Additionally, the To–Te breadth of progesterone-loaded microspheres becomes narrower than that of progesterone-free microspheres. It would be worthwhile to elaborate on the interactions between ethyl formate, progesterone, and PLGA chains through analytical tools such as Fourier transform infrared (FTIR) and Raman spectroscopy.

### 3.5. Thermal Drug Analysis—Contemporary TG Approaches vs. our DTG Technique

Many TG studies qualitatively evaluate drugs in polymeric matrices. For example, Hasseb et al. carried out a TG experiment to demonstrate the presence of chlorhexidine in poly(ethylene glycol)–block–poly(L-lactide) nanoparticles [35]. A TG curve of chlorhexidine-loaded nanoparticles was compared with individual TG curves of chlorhexidine and the copolymer. Elaborating on differences in their mass change profiles, they concluded that chlorhexidine was incorporated into the polymeric nanoparticles. A similar approach was used to assert the encapsulation of ormeloxifine and quercetin in PLGA nanoparticles [36,37]. In fact, their TG curves demonstrated that mass loss occurred between 50 and 100 °C, and slow weight loss continued afterward. However, there was no mention of their causes for such weight loss profiles.

Theoretically, TG can quantify materials of interest in the following cases. First, TG can be used to quantify an inorganic substance loaded into an organic polymeric matrix. For instance, Swy et al. measured the amount of bismuth incorporated into PLGA nanoparticles [38]. Similarly, Saez et al. quantified the platinum-containing oxaliplatin content in PLGA microspheres through a TG experiment [39]. After oxaliplatin-loaded microspheres were fully decomposed, the amount of residual platinum was related to the concentration of oxaliplatin in PLGA microspheres. This approach is based on the principle that residues on ignition are proportional to the concentration of an analyte. Conversely, TG also can quantify an organic compound present in an inorganic material. Nan et al. measured the amount of daunorubicin loaded into porous silicon oxide microparticles, while Zhu et al. determined the content of N-acetyl-cysteine in mesoporus silica nanoparticles [40,41]. However, all these methods belong to non-specific assays. Further, when materials contain unknown impurities, there is always a risk of drawing incorrect conclusions.

**Figure 10:**
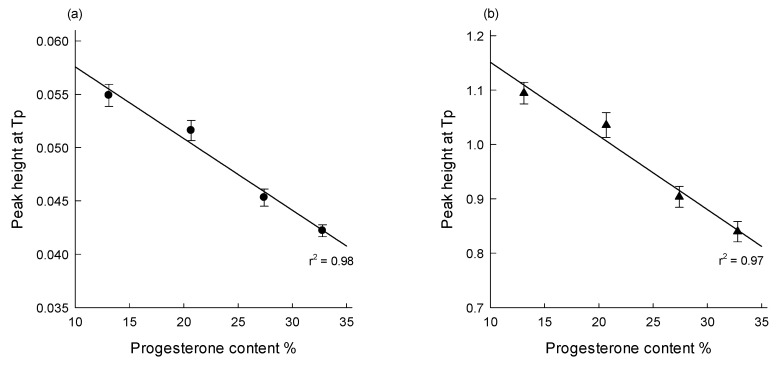
Linear correlation between the peak height at Tp and progesterone payload of microspheres.

Unlike conventional TG practices, we attempted to quantify progesterone using an entirely distinct strategy. The Tp of the progesterone-free microspheres was 89.6 ± 0.5 °C, but PLGA microspheres with different progesterone payloads displayed Tp measures of around 80 °C. As seen in Table 2, there were no significant differences between the results derived from the derivative data of *d*(% mass)/*d*(°C) and those of *d*(% mass)/*d*(min). However, the encapsulation of progesterone into PLGA microspheres led to the reduction of the height of ethyl formate peak at Tp. The higher the actual progesterone payload, the lower the peak height at Tp. As shown in Figure 10, a linear relationship exists between the peak height at Tp and progesterone payload of PLGA microspheres. This result demonstrates that DTG may serve as a convenient alternative to quickly quantify the drug content in microspheres. For example, producing PLGA microspheres with different drug payloads is one of the standard practices used to manipulate the rate and duration of drug release. At this time, their drug contents are first quantified by an analytical tool such as HPLC. The same microspheres are also subject to DTG. Both analytical tools make it possible to establish a correlation between the drug content and the ethyl formate peak. Subsequently, drug contents of various PLGA microspheres manufactured under different conditions can be easily derived simply by monitoring their DTG peak. The drug concentration range will be determined on a case-by-case basis, depending on the targeting duration and rate of drug release.

## 4. Conclusions

The present study reports for the first time the fact that DTG allows for the accurate determination of residual ethyl formate content in PLGA microspheres. Compared to TG, advantages of derivative *d*(% mass)/*d*(min) or *d*(% mass)/*d*(temperature) curves are evident. First, a mass loss event arising from the evolvement of residual ethyl formate is much easier to distinguish. Second, DTG provides detailed information on To and Te temperatures at which residual ethyl formate starts to volatilize and evaporates completely. These parameters allow the accurate quantification of residual ethyl formate in PLGA microspheres. DTG also gives information on the influence of progesterone upon the level of residual ethyl formate, its removal rate, and To–Te breadth. Further, the possibility of quantifying the drug content in PLGA microspheres is suggested on the basis of the linearity between the peak height at Tp and progesterone payload. DTG could be a great alternative to assess residual solvents and drugs in various polymeric matrices, especially in a laboratory environment without GC or TG–EGA.

## Figures and Tables

**Table 1:** Physicochemical properties of ethyl formate and methylene chloride.

Property	Ethyl Formate	Methylene Chloride
Formula	HCOOC_2_H_5_	CH_2_Cl_2_
Molecular mass (g/mol)	74.08	88.93
ICH solvent class	3	2
Boiling point (°C)	54.4	40
Density (g/cm^3^)	0.92	1.33
Solubility in water (g/100 mL)	10.5	1.3
Log P (octanol/water)	0.23	1.25

**Table 2:** Effect of progesterone payload upon the onset, end, and maximum temperatures of ethyl formate peak, as well as its peak area.

Progesterone %	To	Te	Tp	Peak Area
*d*(% mass)/*d*(°C)				
0.0	50.6 ± 0.5	134.4 ± 3.0	89.6 ± 0.5	0.1241 ± 0.0039
13.1	48.0 ± 0.6	121.2 ± 2.6	80.5 ± 0.6	0.0680 ± 0.0033
20.7	50.1 ± 1.0	117.9 ± 2.8	81.6 ± 2.4	0.0628 ± 0.0032
27.4	50.2 ± 0.5	115.2 ± 1.3	80.8 ± 0.9	0.0545 ± 0.0011
32.8	49.3 ± 0.1	116.2 ± 1.1	80.0 ± 0.6	0.0566 ± 0.0015
*d*(% mass)/*d*(min)				
0.0	50.4 ± 0.9	138.8 ± 4.6	89.9 ± 1.3	2.595 ± 0.081
13.1	48.3 ± 0.3	119.1 ± 2.9	80.8 ± 0.8	1.333 ± 0.073
20.7	50.7 ± 1.7	117.0 ± 1.8	82.5 ± 1.9	1.252 ± 0.061
27.4	50.1 ± 1.0	114.7 ± 1.3	81.4 ± 1.2	1.090 ± 0.017
32.8	49.5 ± 0.5	115.0 ± 2.1	79.9 ± 0.9	1.129 ± 0.007

**Table 3:** Comparison of the contents (%) of residual ethyl formate in PLGA microspheres determined by DTG and GC.

Assay	Progesterone Content (%) in Microspheres	
	13.1	32.8
GC	1.93 ± 0.06	1.66 ± 0.06
*d*(% mass)/*d*(°C)	2.13 ± 0.11	1.58 ± 0.02
*d*(% mass)/*d*(min)	2.10 ± 0.11	1.57 ± 0.02

**Table 4:** Comparison of the contents (%) of residual methylene chloride in PLGA microspheres determined by DTG and GC.

Assay	Progesterone Content (%) in Microspheres	
	0	18.5
GC	3.29 ± 0.28	2.77 ± 0.35
DTG, *d*(% mass)/*d*(°C)	3.51 ± 0.20	2.60 ± 0.38

## Supplementary Materials

The following are available online at https://www.mdpi.com/1999-4923/12/7/626/s1, Figure S1. The size distribution patterns of PLGA microspheres prepared by methylene chloride (MC) and ethyl formate (EF). The D_50%_ values of MC microspheres and EF microspheres were 77.4 and 148 μm, respectively. Figure S2. SEM micrographs of (a) blank PLGA microspheres and (b) those with the actual progesterone payload of 18.5%. The microspheres were prepared by the solvent evaporation process using methylene chloride. The size bar is 100 μm. Figure S3. (Left) GC chromatograms of microsphere samples subjected to our GC analytical procedure. Arrows indicate the peaks of ethyl formate (EF), ethyl acetate (EA), and methylene chloride (MC). (Right) Standard calibration curves of the peak areas ratios vs. an analyte (ethyl formate, or methylene chloride) concentration.
