# Supplementary material for: Assessment of Residual Solvent and Drug in PLGA Microspheres by Derivative Thermogravimetry
Source: Pharmaceutics. 2020 Jul 4;12(7):626. doi: 10.3390/pharmaceutics12070626 (PMC7407183; doi:10.3390/pharmaceutics12070626)
Supplement: Supplementary file 1 [file pharmaceutics-12-00626-s001.pdf]

pharmaceutics

Supplementary Materials: Assessment of Residual Solvent and Drug in PLGA Microspheres by Derivative Thermogravimetry

Hyunjin Shim and Hongkee Sah *

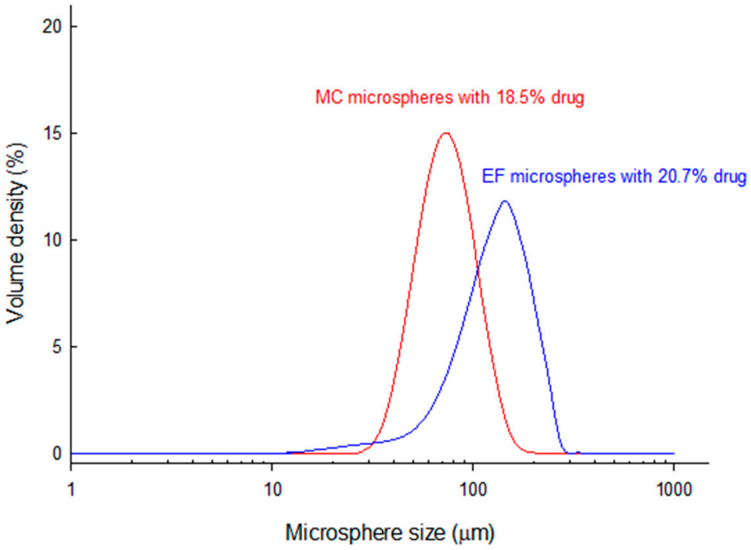

Figure S1. The size distribution patterns of PLGA microspheres prepared by methylene chloride (MC) and ethyl formate (EF). The $D_{50\%}$ values of MC microspheres and EF microspheres were 77.4 and 148 µm, respectively.

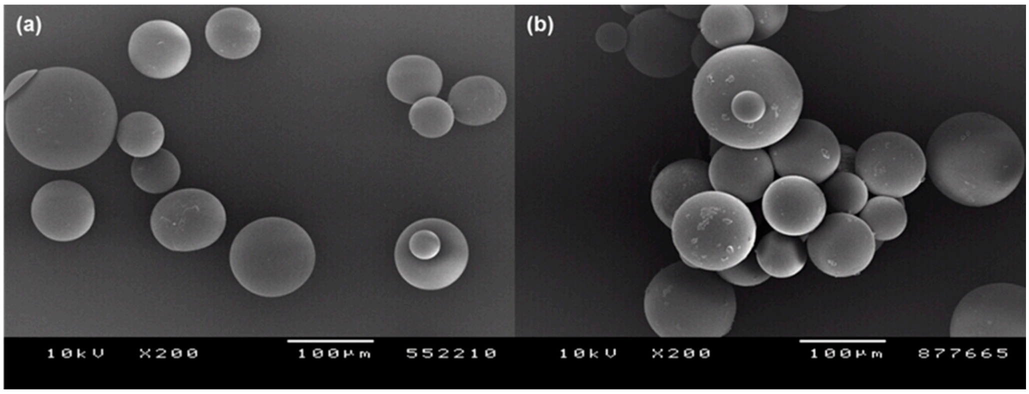

Figure S2. SEM micrographs of (**a**) blank PLGA microspheres and (**b**) those with the actual progesterone payload of 18.5%. The microspheres were prepared by the solvent evaporation process using methylene chloride. The size bar is 100 µm.

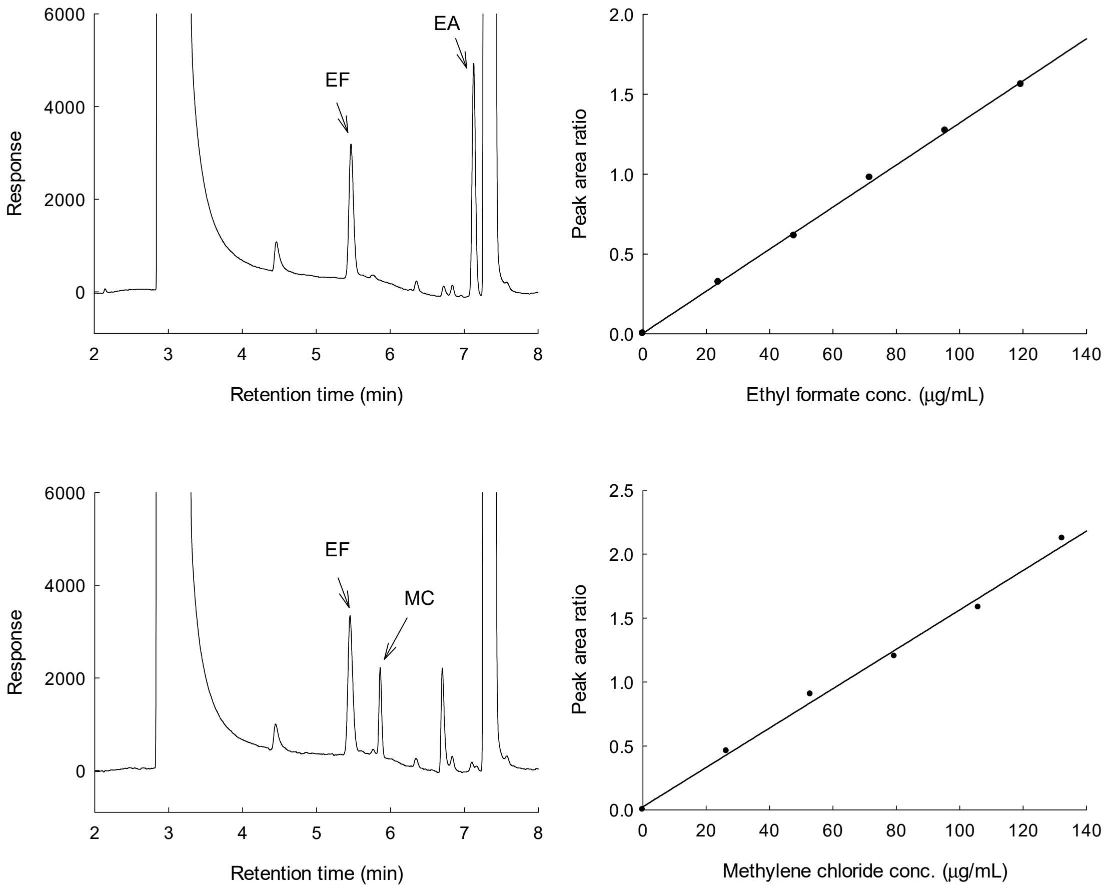

Figure S3. (Left) GC chromatograms of microsphere samples subjected to our GC analytical procedure. Arrows indicate the peaks of ethyl formate (EF), ethyl acetate (EA), and methylene chloride (MC). (Right) Standard calibration curves of the peak areas ratios vs. an analyte (ethyl formate or methylene chloride) concentration.
